# T-type calcium channels functionally interact with spectrin (α/β) and ankyrin B

**DOI:** 10.1186/s13041-018-0368-5

**Published:** 2018-05-02

**Authors:** Agustin Garcia-Caballero, Fang-Xiong Zhang, Victoria Hodgkinson, Junting Huang, Lina Chen, Ivana A. Souza, Stuart Cain, Jennifer Kass, Sascha Alles, Terrance P. Snutch, Gerald W. Zamponi

**Affiliations:** 1 Department of Physiology and Pharmacology, Hotchkiss Brain Institute and Alberta Children’s Hospital Research Institute, Cumming School of Medicine, University of Calgary, 3330 Hospital Dr. NW, Calgary, T2N 4N1 Canada; 2 Michael Smith Laboratories and Djavad Mowafaghian Centre for Brain Health, University of British Colombia, Vancouver, BC Canada

**Keywords:** T-type channels, Spectrin (α/β), Ankyrin B, Trafficking, Cav3.1, Cav3.2

## Abstract

This study describes the functional interaction between the Cav3.1 and Cav3.2 T-type calcium channels and cytoskeletal spectrin (α/β) and ankyrin B proteins. The interactions were identified utilizing a proteomic approach to identify proteins that interact with a conserved negatively charged cytosolic region present in the carboxy-terminus of T-type calcium channels. Deletion of this stretch of amino acids decreased binding of Cav3.1 and Cav3.2 calcium channels to spectrin (α/β) and ankyrin B and notably also reduced T-type whole cell current densities in expression systems. Furthermore, fluorescence recovery after photobleaching analysis of mutant channels lacking the proximal C-terminus region revealed reduced recovery of both Cav3.1 and Cav3.2 mutant channels in hippocampal neurons. Knockdown of spectrin α and ankyrin B decreased the density of endogenous Cav3.2 in hippocampal neurons. These findings reveal spectrin (α/β) / ankyrin B cytoskeletal and signaling proteins as key regulators of T-type calcium channels expressed in the nervous system.

**Electronic supplementary material:**

The online version of this article (10.1186/s13041-018-0368-5) contains supplementary material, which is available to authorized users.

## Introduction

T-type calcium channels are important regulators of neuronal excitability and low threshold-mediated exocytosis [1, 2]. The mammalian genome encodes three different T-type calcium channels (Cav3.1, Cav3.2 and Cav3.3) [3]. The pore forming Cavα1 subunits of these channels are comprised of four homologous transmembrane domains linked by cytoplasmic segments and flanked by intracellular N- and C-terminus structures. T-type channels are highly expressed in the central nervous system including neocortex, cerebellum, thalamus and hippocampus [4] and have been linked to pathophysiologies such as idiopathic generalized epilepsies [5] and tremor [6]. The Cav3.2 subtype is expressed in dorsal root ganglion neurons and spinal cord where its dysregulation contributes to the development of inflammatory and neuropathic pain [7]. Notably, T-type calcium channel blockers are effective in attenuating absence seizures and chronic pain in rodent models [8, 9] and have also been shown efficacious in a human model of inflammatory pain. In this context, understanding how these channels are regulated and trafficked to the cell surface is of importance.

T-type channels are known to interact with numerous regulatory proteins including CamKII [10], G-proteins [11], calcineurin [12], calmodulin [13], syntaxin [1] and calnexin [14], and to form protein complexes with members of the potassium channel family such as Kv4, KCa3.1, and KCa1.1 [15–17]. Many of these interactions appear to involve the cytosolic carboxy-terminal region of Cav3.x proteins and here we utilized a proteomic approach to identify additional interacting partners. In particular, we focused on a stretch of conserved negatively charged residues located in the proximal carboxy-terminal regions of Cav3.1 and Cav3.2 channels. Mass spectrometry identified spectrins as Cav3.2 C-terminal interacting proteins. We further describe the interactions and functional regulation of Cav3.1 and Cav3.2 channels with spectrin (α/β) and ankyrin B in both exogenous expression and native systems, revealing that these interactions regulate whole cell current density. Together, the results indicate that cytoskeletal elements are important regulators of T-type calcium channel function and physiology.

## Results

### Spectrins interact with T-type calcium channels

**Fig. 1:**
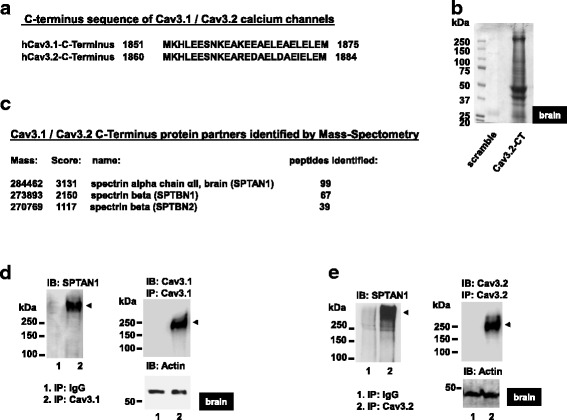
Identification of spectrin (a/β) as a Cav3.1 / Cav3.2 calcium channel interacting protein. (**a**) Conserved proximal C-terminus region of Cav3.1 and Cav3.2 calcium channels. (**b**) Proteins bound to scramble (lane 1) or Cav3.2 CT 1860–1884 biotinylated peptides (lane 2) from mouse whole brain lysates, as seen by Coomassie staining (**c**) Mass spectrometry analysis of proteins bound to the Cav3.2 CT 1860–1884 biotinylated peptide by affinity precipitation assay using mouse whole brain lysates. (**d**) Cav3.1 or (**e**) Cav3.2 immunoprecipitates from mouse whole brain lysates were probed for spectrin αII (SPTAN1) by Western blot. An actin loading control is shown

The proximal C-terminus regions of Cav3.1 and Cav3.2 contain a conserved α-helical (http://bioinf.cs.ucl.ac.uk/psipred) stretch of charged amino acid residues (amino acids 1851–1875 in Cav3.1, and 1860–1884 in Cav3.2) (Fig. 1a). To determine whether this motif is a binding region for regulatory proteins, we used a proteomic approach with a corresponding Cav3.2 CT synthetic peptide conjugated with biotin as bait for interacting proteins in mouse brain lysates. Bound proteins were resolved in a denaturing Coomassie gel (Fig. 1b) and protein bands that appeared in samples incubated with the Cav3.2CT bait, but not in samples incubated with the control scramble peptide, were excised and analyzed by MALDI/TOF mass spectrometry. This analysis yielded hits for three cytoskeletal proteins: spectrin- αII (SPTAN1) and two isoforms of spectrin-β (SPTBN1 and SPTBN2) (Fig. 1c), with the former showing the highest score. Spectrin is a heterodimeric protein comprised of α- and β- subunits (280 and 246 kDa, respectively) that form a supercoiled triple-helix structure through 106 residue modules known as “spectrin repeats” [18]. This particular structural organization allows spectrin to expand and contract to remodel the cytoskeleton and, hence, cellular architecture [19]. In humans, there are two α and five β spectrin subunit genes. Spectrin-αI is expressed in erythroid cells whereas spectrin-αII is expressed in all nonerythroid cells, including the brain where it is important for synaptic transmission [20] and participates in neurotransmitter release [21]. Spectrins possess multiple interacting domains such as a pleckstrin homology domain, a Src homology 3 domain (SH3), a calcium binding EF hand domain, an ankyrin binding repeat and an actin binding domain, that interact with a diverse set of cell signaling proteins, receptors and ion channels [18]. To confirm SPTAN1-Cav3 interactions, we performed co-immunoprecipitations between SPTAN1 and either Cav3.1 or Cav3.2. Figure 1d and e show that SPTAN1 co-immunoprecipitated with both T-type channel isoforms from mouse brain lysates.

**Fig. 2:**
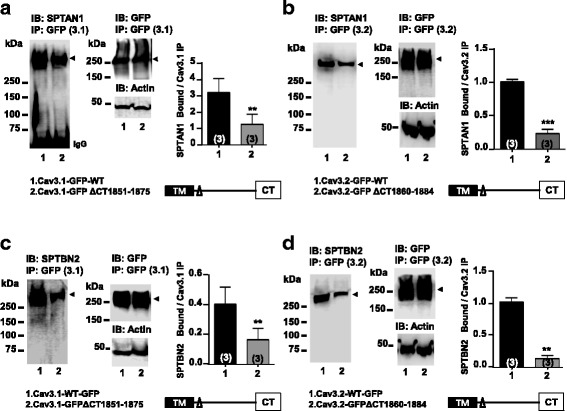
Effect of Cav3.1-GFP and Cav3.2-GFP C-terminal deletions on SPTAN1 and SPTBN2 binding in tsA-201 cells. (**a**) Cav3.1-GFP ∆CT (1851–1875) and wild type channel immunoprecipitates probed with anti-Spectrin αII (SPTAN1) polyclonal antibody. Densitometry analysis of SPTAN1 bound to Cav3.1-GFP immunoprecipitates is shown. (*P* = 0.0051, *n* = 3). (**b**) Cav3.2-GFP ∆CT (1860–1884) and wild type channel immunoprecipitates probed with anti-Spectrin αII (SPTAN1) polyclonal antibody. Densitometry analysis of SPTAN1 bound to Cav3.2-GFP immunoprecipitates is shown (*P* = 0.0005, n = 3). (**c**) Cav3.1-GFP ∆CT (1851–1875) and wild type channel immunoprecipitates probed with anti-Spectrin βII (SPTBN2) polyclonal antibody. Densitometry analysis of SPTBN2 bound to Cav3.1-GFP immunoprecipitates is shown (*P* = 0.0085, n = 3). (**d**) Cav3.2-GFP ∆CT (1860–1884) and wild type channel immunoprecipitates probed with anti-Spectrin βII (SPTBN2) polyclonal antibody. Densitometry analysis of SPTBN2 bound to Cav3.2-GFP immunoprecipitates is shown. (*P* = 0.0047, n = 3)

We next asked whether deletion of the helical region in the proximal C-terminus regions of Cav3.1 and Cav3.2 could alter their association with SPTAN1. For this purpose, we expressed GFP-tagged wild type or deletion-mutant channels in tsA-201 cells and performed co-immunoprecipitations between the exogenously expressed channels and endogenous spectrin. Consistent with the data in Fig. 1, both GFP-tagged Cav3.1 and Cav3.2 channels co-immunoprecipitated with SPTAN1 (Fig. 2a and b). Deleting the helical region (Cav3.1-GFP ∆CT (1851–1875), Cav3.2-GFP ∆CT (1860–1884)) reduced, but did not completely eliminate binding of SPTAN1 to both channel isoforms (Fig. 2a and b), confirming that this region is involved in spectrin interactions, but also that spectrin may associate with contact sites in other regions of the channels. These putative additional interactions do not appear to involve the distal C-terminus regions, since deletion of residues 1875–2377 in Cav3.1 (Cav3.1-GFP ∆CT (1875–2377)) had no effect on co-immunoprecipitations with SPTAN1 (Additional file 1: Figure S1).

Given that other spectrin isoforms were pulled down with the C-terminal helix bait, we wanted to confirm these interactions in tsA-201 cells. Unfortunately, these cells do not appear to express spectrin-β1, and hence we could not test these interactions with this expression system. However, tsA-201 cells do express spectrin-βII (SPTBN2) which could be co-immunoprecipitated with both Cav3.1-GFP and Cav3.2-GFP (Fig. 2c and d). As with SPTAN1, the SPTBN2 interactions were significantly weakened by deleting the helical regions in the Cav3.1 and Cav3.2 C-termini (Fig. 2c and d). As these data are based upon co-immunoprecipitations and that SPTAN1 and SPTBN2 interact with each other, it is unclear if either the two spectrin isoforms is the primary interaction partner, or whether there may be another intermediate protein(s) involved in linking the channels to the cytoskeleton. Taken together, these data indicate that spectrins physically interact with a putative helical region conserved in the C-terminal domains of Cav3.1 and Cav3.2 calcium channels.

### Ankyrin B interacts with T-type calcium channels

**Fig. 3:**
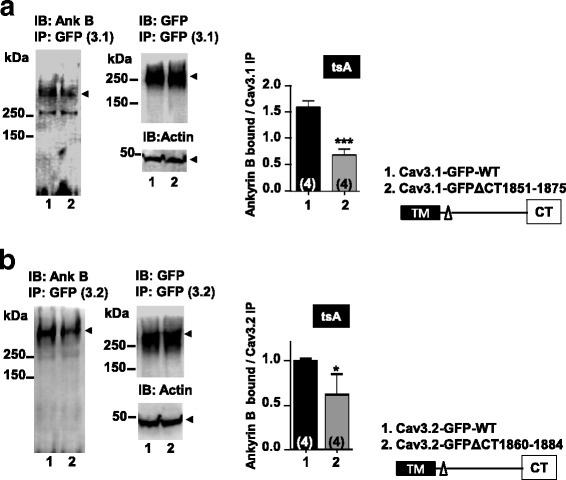
Effect of Cav3.1-GFP and Cav3.2-GFP C-terminal deletions on ankyrin B binding in tsA-201 cells. (**a**) Cav3.1-GFP ∆CT (1851–1875) and wild type channel immunoprecipitates probed with anti-ankyrin B polyclonal antibody. Densitometry analysis of ankyrin B bound to Cav3.1-GFP immunoprecipitates is shown (*P* = 0.0005, *n* = 4). (**b**) Cav3.2-GFP ∆CT (1860–1884) and wild type channel immunoprecipitates probed with anti-ankyrin B polyclonal antibody. Densitometry analysis of ankyrin B bound to Cav3.2-GFP immunoprecipitates is shown (*P* = 0.015, n = 4)

Direct interactions with spectrin have been reported for epithelial sodium channels, whereas neuronal voltage-gated sodium channels are reportedly linked to spectrin via ankyrin B or G [22]. We therefore examined binding of ankyrin B to Cav3.1 and Cav3.2 channels. Following expression of Cav3.1-GFP and Cav3.2-GFP constructs in tsA-201 cells immunoprecipitates with GFP were probed with an ankyrin B antibody, revealing interactions with both channel isoforms **(**Fig. 3a and b). Deletion of the proximal C-terminal alpha helix in the two channels reduced ankyrin B interactions with Cav3.1 and to a lesser extent with Cav3.2 (Fig. 3a and b). These data raise the possibility that, similar to voltage gated sodium channels, spectrin may link to Cav3 channels via ankyrin interactions.

### Functional role of the Cav3 cytoskeletal interacting domain

**Fig. 4:**
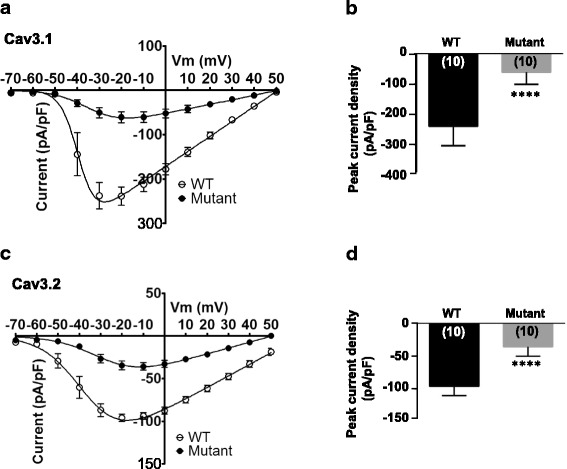
Calcium currents evoked by wild type and mutant Cav3.1 and Cav3.2 channels in tsA-201 cells. (**a**) Current-voltage (I/V) relationship of wild type or mutant Cav3.1-GFP ∆CT (1851–1875) mutant channels (*n* = 10). Values are represented as means +/− S.E. The solid lines are fits with the Boltzmann equation. (**b**) Mean peak current density representation from wild type or mutant Cav3.1-GFP ∆CT (1851–1875) channels (*P* < 0.001, n = 10). Asterisks denote statistical significance relative to wild type (****P < 0.0001, Student’s t-test). (**c**) Current-voltage (I/V) relationship of wild type or mutant Cav3.2-GFP ∆CT (1860–1884) channels (*n* = 18–19). Values are represented as means +/− S.E. The solid lines are fits with the Boltzmann equation. (**d**) Mean peak current density representation from wild type or mutant Cav3.2-GFP ∆CT (1860–1884) channels (P < 0.001, n = 10). Asterisks denote statistical significance relative to wild type (****P < 0.0001, Student’s t-test)

We next examined whole cell calcium currents from wild type and deletion-mutant Cav3.1 expressed in tsA-201 cells by recording current-voltage relationships for wildtype Cav3.1 (wt) and mutant Cav3.1 (∆1851–1875). Deletion of (MKHLEESNKEAKEEAELEAELELE) reduced Cav3.1 current density by approximately 75% (wt Cav3.1 = − 238.68 ± 20.36 pA/pF at − 20 mV; Cav3.1 ∆1851–1875 = − 60.96 ± 12.71 pA/pF at − 20 mV; *P* < 0.001, *n* = 10); Fig. 4a and b). Similarly, for wt Cav3.2 and mutant Cav3.2 (∆1860–1884) deletion of conserved sequence (MKHLEESNKEAREDAELDAEIELE) significantly reduced the current density of Cav3.2 (wt = − 95.84 ± 4.45 pA/pF at − 20 mV; Cav3.2 ∆1860–1884 = − 35.67 ± 4.60 pA/pF at − 20 mV; P < 0.001, n = 10; Fig. 4c and d). There were no changes in the voltage-dependence of activation of the channels, nor were there any changes in channel kinetics (not shown), suggesting that the reduced current densities might be due to fewer channels in the plasma membrane. This notion is supported by experiments examining the cell surface pool of transiently expressed Cav3.1 channels incubated with a cell permeant (i.e., Tat epitope fused) disruptor peptide corresponding to the putative spectrin interaction site. When compared to a scrambled Tat control peptide, Cav3.1 cell surface expression was diminished by the disruptor peptide (Additional file 1: Figure S2), indicating that the cytoskeletal interactions may regulate the cell surface density of Cav3.1 channels.

**Fig. 5:**
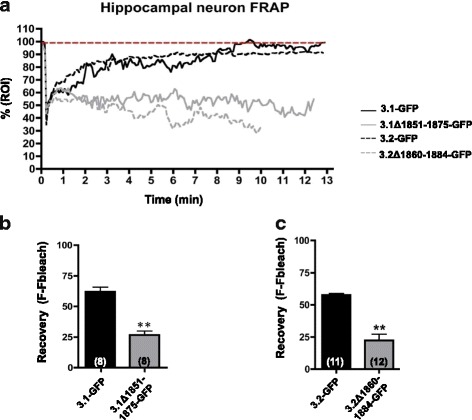
Mobility of wild type and mutant Cav3.1 and Cav3.2 channels in hippocampal neurons. (**a**) Fluorescence recovery after photobleaching (FRAP) assay of Cav3.1-GFP wild type and ∆CT (1851–1875) mutant channels or Cav3.2-GFP wild type and ∆CT (1860–1884) mutant channels transfected into mouse hippocampal neuron cultures. (**b**) Recovery (Fluorescence-Fluorescence bleach) of Cav3.1-GFP wild type and ∆CT (1851–1875) mutant channels**.** Recovery values are calculated as F(maximum after photobleach)-F(photobleach) (*n* = 8 WT, n = 8 mutant, *P* < 0.01, Student’s t-test). (**c**) Recovery (Fluorescence-Fluorescence bleach) of Cav3.2-GFP wild type and ∆CT (1860–1884) mutant channels**.** Recovery values are calculated as F(maximum after photobleach)-F(photobleach) (*n* = 11 WT, *n* = 12 mutant, *P* < 0.01, Student’s t-test)

Next, we tested whether deletion of the cytoskeleton interaction region in Cav3 channels altered Cav3.x channel trafficking. To address this, we transfected full-length and deletion-mutant Cav3-GFP channels into cultured hippocampal neurons and performed fluorescence recovery after photobleaching (FRAP) experiments. Fluorescence intensity mediated by the full-length Cav3.1-GFP and Cav3.2-GFP channels was found to recover more strongly than signals mediated by the deletion mutants (Fig. 5a-c), suggesting that the reduced ability of the deletion mutants to interact with cytoskeletal proteins affects the lateral mobility or insertion of new Cav3 channels in the plasma membrane.

**Fig. 6:**
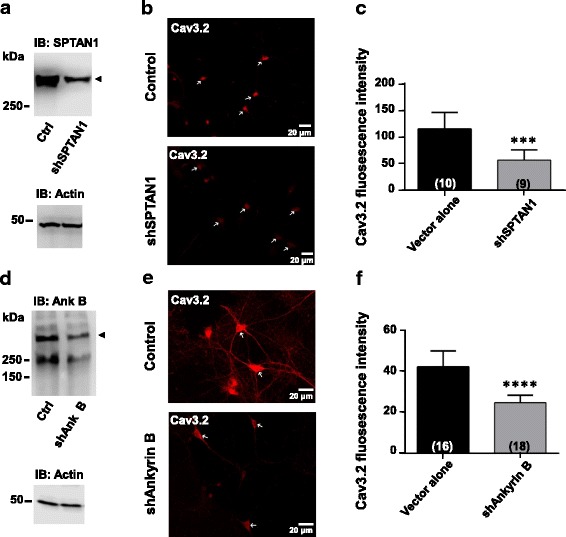
SPTAN1 and ankyrin B knockdown effect on endogenous Cav3.2 calcium channel density in mouse and rat hippocampal neurons. (**a**) SPTAN1 expression levels in mouse hippocampal neurons treated with shRNA as seen by western blot. (**b**) Mouse hippocampal neurons untreated or treated with shRNA for SPTAN1 were stained for Cav3.2 channels with a specific anti-Cav3.2 polyclonal antibody. (**c**) Cav3.2 channel intensity values from untreated neurons or neurons treated with SPTAN1 shRNA. (**d**) Ankyrin B expression levels in rat hippocampal neurons treated with shRNA as seen by western blot. (**e**) Rat hippocampal neurons untreated or treated with shRNA for ankyrin B were stained for Cav3.2 channels with a specific anti-Cav3.2 polyclonal antibody. (**f**) Cav3.2 channel intensity values from untreated neurons or neurons treated with specific ankyrin B shRNA

We then examined the expression of endogenous Cav3 channels in cultured hippocampal neurons in response to knockdown of SPTAN1 and ankyrin B. For these experiments we focused on the Cav3.2 isoform. Western blots confirmed the expression and subsequent shRNA-mediated knockdown of endogenous SPTAN1 and ankyrin B in hippocampal neurons (Fig. 6a and d). We performed immunostaining experiments in which endogenous Cav3.2 channels were stained with a Cav3.2 antibody. Cav3.2 channel expression was evident in cell bodies, axons and proximal dendrites (Fig. 6b and e). Upon shRNA-mediated knockdown of SPTAN1, total Cav3.2 fluorescence was consistently diminished (Fig. 6b and c). A similar effect was observed upon knockdown of ankyrin B (Fig. 6e and f). Altogether, these data are consistent with the reduced channel current densities and FRAP signals observed upon depletion of the Cav3 interaction domain, and support an effect of cytoskeletal elements in the expression of native T-type calcium channels.

## Discussion

In this study, we used a proteomic approach to identify interacting partners of the proximal Cav3.1 and Cav3.2 C-terminus regions. The screen identified both α and β spectrin as both interacting partner with the first 24 amino acids of the C terminal region. Both spectrin subtypes are membrane cytoskeleton components [23, 24] and there is a growing body of literature implicating spectrins in the clustering of ion channels (e.g., sodium and potassium channels) at specific subcellular loci [25–27], often indirectly via specific ankyrins [22, 28, 29]. We confirmed the spectrin interactions via co-immunoprecipitation from mouse brain tissue and also identified ankyrin B as part of the putative Cav3.2/spectrin binding complex. Deletion of 24 amino acid residues of the proximal Cav3 C-terminus from the full-length channels reduced whole cell current densities in transient expression systems. Furthermore, shRNA depletion of both spectrin α and ankyrin B in hippocampal neurons reduced Cav3.2 type channel expression. Finally, fluorescence recovery experiments using GFP-tagged Cav3 channels in hippocampal neurons revealed that Cav3.1 and Cav3.2 channels lacking the interaction domain display significantly reduced mobility in the plasma membrane and/or trafficking to the membrane compared to wild type channels. Taken together, we conclude that cytoskeleton interactions are important determinants of T-type calcium channel trafficking to and within the plasma membrane. The molecular mechanisms by which these cytoskeletal interactions affect T-type channel trafficking remain to be determined. Within the plasma membrane, interactions of the channel with the cytoskeleton may facilitate the lateral trafficking due to dynamic cytoskeletal rearrangements. It is also possible that interactions with spectrin facilitate the effective translocation of the channel from the endoplasmic reticulum to the cell surface, either by facilitating transport, or by occluding an ER retention signal.

The data indicate that the proximal carboxy-terminus regions of Cav3.1 and Cav3.2 binds to spectrins (α/β) via a 24 amino acid stretch containing many negatively charged residues located in close proximity to the transmembrane domain of these channels. This interaction may be responsible for promoting binding of ankyrin B to the complex, likely via direct binding to the described α/β spectrin ankyrin binding repeat [18]. In contrast with the previously identified proline rich motif present in other ion channels [30] that binds to the SH3 domain in spectrins, this novel motif conserved across T-type calcium channels is enriched with glutamic and aspartic acids, possibly involved in electrostatic interactions with spectrin (α/β).

Literature from the voltage-gated sodium channel field identifies ankyrin as a key determinant of sodium channel clustering at Nodes of Ranvier [31, 32]. It is thus possible that spectrin/ankyrin interactions with Cav3 mediate a similar clustering/targeting role for localization of T-type channels at specific subcellular loci. The observation that deletion of the putative spectrin/ankyrin interaction motif in both Cav3.1 and Cav3.2 channels greatly impeded fluorescence recovery in FRAP experiments and reduced whole cell current densities is consistent with a role of the cytoskeleton trafficking to and within the plasma membrane. Whether these interactions are involved in targeting the channels to specific membrane compartments such as nodal regions, or synaptic/dendritic sites remains to be determined.

In the nervous system, T-type calcium channels fulfill two major roles. One is to regulate the excitability of neurons, being of particular importance in the corticothalamic circuitry wherein T-type channels are known to mediate rebound bursting [33] and is of particular relevance to the genesis of absence seizures [34]. A second major role that has emerged more recently is their contribution towards low threshold-mediated neurotransmitter release [1]. Indeed, Cav3 channels interact with syntaxin 1A and have been shown to contribute to synaptic release in dorsal horn synapses [7, 35, 36]. This is of particular relevance for conditions such as neuropathic pain where increases in Cav3 channel current density facilitate neuronal firing and synaptic communications in the afferent pain pathway. Given their potent effects on T-type channel expression, spectrins and ankyrins may act as regulatory elements for controlling nervous system function via T-type channel interactions.

The regulation of T-type calcium channels by cytoskeletal elements is likely to extend beyond the nervous system. For example, ankyrin B is highly expressed in cardiac myocytes where it regulates excitation-contraction coupling in concert with other signaling molecules [37]. Myocytes also express Cav3.1 and Cav3.2 T-type calcium channels, especially during early post-natal development [38]. T-type channel expression is however increased under pathological conditions such as cardiac hypertrophy [39] thus it would be interesting to determine whether T-type channel expression increases are related to cytoskeletal remodeling.

## Conclusion

In summary, we have identified cytoskeletal interactions as a molecular mechanism for regulating T-type channel mobility and current density. It is possible that targeting these interactions may offer a means for affecting T-type calcium channel activity in disorders such as chronic pain and epilepsy.

## Methods

### Drugs and peptides

Human biotin-Cav3.2-CT 1860–1884, biotin-Cav3.2-CT scramble peptides (Genemed synthesis, San Antonio, Tx). SPTAN1 and ankyrin B shRNAs were purchased from Thermo Scientific, Open Biosystems.

### Cell culture and transfection

Human embryonic kidney tsA-201 cells were cultured as described [40]. Cells were transfected with Lipofectamine 2000 and used for biochemical and electrophysiological analysis 48–72 h post-transfection.

### Hippocampal neuron primary cultures

Mouse or rat hippocampal neurons were dissociated as described before [41] and seeded at low density onto coverslips pretreated with poly-D-lysine (Sigma) followed by Laminin (Sigma) in 24-well plates. At day six of culture, transfection of cDNA was performed using Lipofectamine 2000 (Invitrogen) following the manufacturer’s instructions. We used 1.5 μg of cDNA per well with 2 μl of Lipofectamine. cDNA and Lipofectamine solution were mixed together for 30 min at room temperature. Cells were incubated in cDNA–Lipofectamine 2000 complexes for 2 h at 37 °C and coverslips were placed back in their medium. Four days after transfection, immunostaining with GFP antibody (1500) was conducted at 37 °C.

### Plasmids

To generate the GFP-tagged Cav3.1 or Cav3.2, the coding sequences of human Cav3.1 or human Cav3.2 was cloned into the pcDNA3.1(+) vector (Invitrogen) with stop codon removed; GFP was amplified by PCR and inserted into the C-terminus of Cav3.1 or Cav3.2. To delete the specific fragment amino acids 1860–1884 from Cav3.1 or Cav3.2, we used two-step PCR. The first round of PCR amplified the upstream and downstream flanking regions of that fragment. Products were purified and used as templates for the second round of PCR, which recombined these two regions together. The final PCR product was used to replace wide type Cav3.1 or Cav3.2 by making use of appropriate restriction sites in the two flanking regions.

### Affinity precipitation of Cav3.2 interacting proteins

Mouse brain proteins were solubilized in buffer (in mM; 50 Tris pH 7.6, 150 NaCl, 1% Triton X-100, 1% NP40, 10 EDTA, 10 EGTA and protease inhibitors). Soluble proteins were collected by centrifugation at 16,100 g for 10 min. Supernatant fractions (500 μg) were precleared by incubation with neutravidin beads for 1 h at 4 °C (Thermo Scientific) and then incubated in a modified solubilization buffer (in mM; 50 Tris pH 7.6, 150 NaCl, 0.2% Triton X-100, 0.2% NP40, 10 EDTA, 10 EGTA and protease inhibitors) for 2 h at 4 °C with a human Cav3.2-carboxy-terminal (1860–1884 a.a.(MKHLEESNKEAREDAELDAEIELEM)) or a scramble (SEMADLEKAENHMDEIMEKAEEREL) biotinylated peptides (5 μg) covalently linked to a C-terminal biotin (Genemed synthesis Inc., San Antonio, Tx). After Cav3.2-interacting proteins were collected, samples were washed three times with modified solubilization buffer. Bound proteins were analyzed by SDS-PAGE and visualized by Coomassie blue staining (Sigma). Visible bands were excised and samples analyzed by MALDI/TOF-MS (Bruker Instruments Co., Bremen, Germany).

### Western blotting

Western blot analysis was performed using anti-actin mouse (Sigma), anti-GFP (Abcam) anti-αII-spectrin (Santa Cruz, Biotechnology, Inc.) and anti-ankyrin B (Santa Cruz Biotechnology, Inc.) rabbit antibodies. Western blot quantification was performed using densitometry analysis (Quantity One-BioRad software). Student’s t-tests for unpaired data were performed to determine statistical significance.

### Co-immunoprecipitation assays

Mouse whole brain tissue or tsA-201 cells were lysed in a modified RIPA buffer (in mM; 50 Tris, 100 NaCl, 0.2% (*v*/v) Triton X-100, 0.2% (v/v) NP-40, 10 EDTA + protease inhibitor cocktail, pH 7.5) that was used to co-immunoprecipitate Cav3.2 channels with spectrin (α/β) or ankyrin B proteins. Lysates were prepared by sonicating samples at 60% pulse for 10 s and by centrifugation at 13,000 rpm for 15 min at 4 °C. Supernatants were transferred to new tubes and solubilized proteins were incubated with 50 μl of Protein G/A beads (Piercenet) and 2 μg of anti-Cav3.2 (H-300, Santa Cruz Biotechnologies, Inc) antibody or anti-GFP antibody (Abcam) overnight while tumbling at 4 °C. Total inputs were taken from whole cell samples representing 4% of total protein and probed for actin. Co-immunoprecipitates were washed twice with (mM) 150 NaCl 50 Tris pH 7.5 buffer, beads were aspirated to dryness. Laemmli buffer was added and samples were incubated at 96 °C for 7 min. Eluted samples were loaded on 7.5% Tris-glycine gel and resolved using SDS-PAGE. Samples were transferred to 0.45 mm polyvinylidenedifluoride (PDVF) membranes by dry transfer using an Iblot machine (Invitrogen).

### Electrophysiological recordings

Whole-cell voltage-clamp recordings for tsA-201 cells were performed 72 h after transfection with cDNA (Cav3.1-GFP wild type (2 μg) or Cav3.1-GFP Mutant (2 μg) and Cav3.2-GFP wild type or Cav3.2-GFP Mutant (2 μg)). Recordings were conducted with 10 mM barium as the charge carrier, using internal and external recording solutions; (in mM): 110 CsCl, 3 Mg-ATP, 0.5 Na-GTP, 2.5 MgCl2, 5 D-glucose, 10 EGTA, 10 HEPES (pH 7.3 with CsOH). The external solution contained (in mM): 10 BaCl2, 1 MgCl2, 140 TEACl, 10 D-glucose, 10 HEPES (pH 7.2 with TEAOH). Currents were elicited from a holding potential of − 100 mV and depolarized from − 70 to + 50 mV with 10-mV increments. Data were collected from multiple batches of transfections with similar numbers of cells tested from the different groups. For data analysis, peak currents and cell capacitance were measured and converted into current density.

### FRAP assays

Primary hippocampal neurons were transfected at 10 DIV and imaged 48 h afterward. Transfections were done with 2 μg of DNA/ matek dish with 6ul of Lipofectamine 2000 for 2 h.

Photobleaching experiments: FRAP experiments were imaged using a ZEISS 510 LSM. A ROI was selected on an axon proximal to the cell body of a neuron. After 3 baseline images, 10 iterations of 100% laser power was used to bleach the ROI, and recovery imaged for 13 min, with a picture every 5 s. Fluorescence intensity values were normalized to an initial intensity of 100%. Cells were excluded if photobleaching did not decrease fluorescence intensity to more than 50% of initial 100%. Traces: Fluorescence intensity values are normalized to an initial value of 100%. Intensity values were normalized to an initial value of 100%. Recovery values are calculated as F maximum after photobleach- F photobleach.

### Immunofluorescence

Briefly, cultured rat hippocampal neurons were washed twice with PBS containing (mM) 1 MgCl_2_ and 2 CaCl_2_. Neurons were fixed with 4% paraformaldehyde for 20 min at room temperature (RT) and washed 3 times after fixation. Then neurons were permeabilized with PBS containing 0.1% triton x-100 and 2 mg/ml BSA for 30 min. Neurons were blocked with PBS containing 4% milk and 2 mg/ml BSA for 2 h at RT. Cav3.2 (mouse anti-Cav3.2, 1 μg/ml, Novus Biologicals,CA) primary antibodies were incubated overnight at 4 °C. After washing three times, the secondary antibodies Alexafluor 546 conjugated Donkey anti-mouse (Thermo Fisher Scientific, CA) were incubated 2 h. All images were digitally captured with an 8 bit camera, thus giving grey level (intensity) values of 0–255. Immunostaining was visualized using a 40 × 0.4 NA objective lens on a Zeiss LSM 510 META confocal systems, running Velocity 6.

### Data analysis and statistics

For biochemical and electrophysiological analyses, data values are presented as mean ± SEM for n experiments. Statistical significance was determined using Student’s t test unless stated otherwise: *p < 0.05; ** *p* < 0.01; *** *p* < 0.001; NS, statistically not different.

## Additional file

Additional file 1: **Figure S1.** Binding of SPTAN1 to Cav3.1-GFP ΔCT 1875–2377 mutant channels lacking a distal C-terminus region. Cav3.1-GFP ∆CT (1875–2377) and wild type channel immunoprecipitates from transfected tsA-201 cells probed with anti-Spectrin αII (SPTAN1) polyclonal antibody. Densitometry analysis of SPTAN1 bound to Cav3.1-GFP immunoprecipitates is shown. **Figure S2.** Disrupting Cav3.1 SPTAN1 interactions reduces cell surface expression of Cav3.1. Left: Surface biotinylation experiments on Cav3.1 channels transiently expressed in tsA-201 cells in the presence of a cell permeant Tat peptide corresponding to the putative spectrin interaction site (Tat-Cav3.1-CT) on the channel, or a scrambled peptide sequence. Right: Densitometry analysis of Cav3.1 surface pool normalized to the actin control. Note that the Tat- Cav3.1-CT peptide reduces the cell surface expression of the channel by ~ 40%. (DOC 118 kb)

## Abbreviations

FRAP: Fluorescence recovery after photobleaching
GFP: Green fluorescent protein
PCR: Polymerase chain reaction
shRNA: Short hairpin ribonucleic acid, wt: wild type
