# Supplementary material for: T-type calcium channels functionally interact with spectrin (α/β) and ankyrin B
Source: Mol Brain. 2018 May 2;11:24. doi: 10.1186/s13041-018-0368-5 (PMC5930937; doi:10.1186/s13041-018-0368-5)
Supplement: Supplementary file 1 — Figure S1. Binding of SPTAN1 to Cav3.1-GFP ΔCT 1875–2377 mutant channels lacking a distal C-terminus region. Cav3.1-GFP ∆CT (1875–2377) and wild type channel immunoprecipitates from transfected tsA-201 cells probed with anti-Spectrin αII (SPTAN1) polyclonal antibody. Densitometry analysis of SPTAN1 bound to Cav3.1-GFP immunoprecipitates is shown. Figure S2. Disrupting Cav3.1 SPTAN1 interactions reduces cell surface expression of Cav3.1. Left: Surface biotinylation experiments on Cav3.1 channels transiently expressed in tsA-201 cells in the presence of a cell permeant Tat peptide corresponding to the putative spectrin interaction site (Tat-Cav3.1-CT) on the channel, or a scrambled peptide sequence. Right: Densitometry analysis of Cav3.1 surface pool normalized to the actin control. Note that the Tat- Cav3.1-CT peptide reduces the cell surface expression of the channel by ~ 40%. (DOC 118 kb) [file 13041_2018_368_MOESM1_ESM.doc]

**Additional File 1 – Supplemental Data**

***
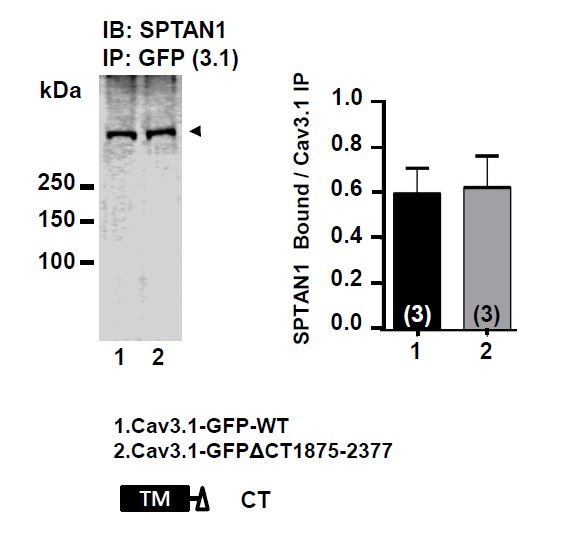
***

***Fig. S1. Binding of SPTAN1 to Cav3.1-GFP ΔCT 1875-2377 mutant channels lacking a distal C-terminus region.***

Cav3.1-GFP ∆CT (1875-2377) and wild type channel immunoprecipitates from transfected tsA-201 cells probed with anti-Spectrin αII (SPTAN1) polyclonal antibody. Densitometry analysis of SPTAN1 bound to Cav3.1-GFP immunoprecipitates is shown.


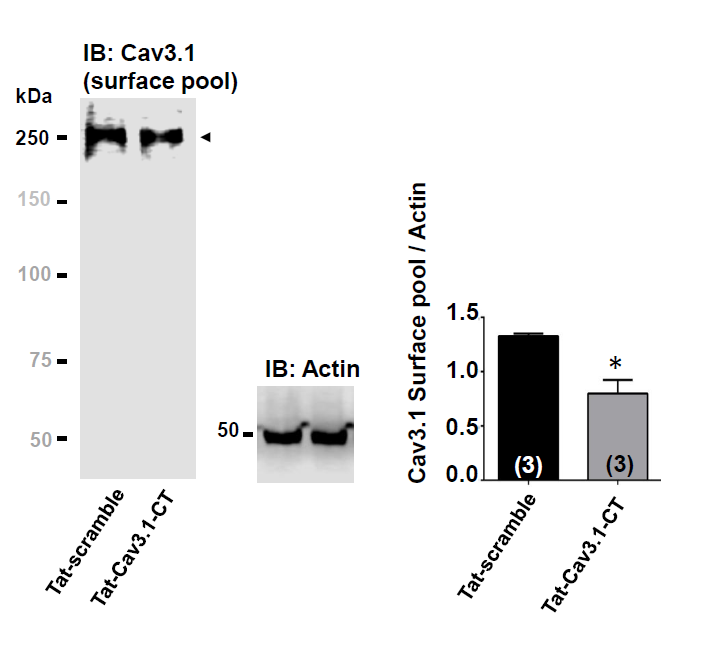


***Fig. S2.Disrupting Cav3.1 SPTAN1 interactions reduces cell surface expression of Cav3.1***

Left: Surface biotinylation experiments on Cav3.1 channels transiently expressed in tsA-201 cells in the presence of a cell permeant Tat peptide corresponding to the putative spectrin interaction site (Tat-Cav3.1-CT) on the channel, or a scrambled peptide sequence. Right: Densitometry analysis of Cav3.1 surface pool normalized to the actin control. Note that the Tat- Cav3.1-CT peptide reduces the cell surface expression of the channel by ~40%.
